# Redundant Mechanisms for Regulation of Midline Crossing in *Drosophila*

**DOI:** 10.1371/journal.pone.0003798

**Published:** 2008-11-24

**Authors:** Giorgio F. Gilestro

**Affiliations:** Research Institute of Molecular Pathology (IMP), Vienna, Austria; Institut Pasteur, France

## Abstract

During development, all neurons have to decide on whether to cross the longitudinal midline to project on the contralateral side of the body. In vertebrates and invertebrates regulation of crossing is achieved by interfering with Robo signalling either through sorting and degradation of the receptor, in flies, or through silencing of its repulsive activity, in vertebrates. Here I show that in *Drosophila* a second mechanism of regulation exists that is independent from sorting. Using in vitro and in vivo assays I mapped the region of Robo that is sufficient and required for its interaction with Comm, its sorting receptor. By modifying that region, I generated new forms of Robo that are insensitive to Comm sorting in vitro and in vivo, yet still able to normally translate repulsive activity in vivo. Using gene targeting by homologous recombination I created new conditional alleles of *robo* that are sorting defective (*robo^SD^*). Surprisingly, expression of these modified proteins results in phenotypically normal flies, unveiling a sorting independent mechanism of regulation.

## Introduction

The nervous system of all bilaterally symmetric organisms is composed of two populations of neurons: ipsilateral and contralateral. Ipsilateral neurons connect to synaptic targets that lie on the same side of the body, while contralateral neurons extend their axons across the midline to contact targets located on the opposite side of the body. The decision of whether to cross the midline is universal (as it is a decision every neuron has to make), binary and irreversible. These characteristics make it an extremely interesting biological model for developmental neurobiologists, so much that it is arguably the best studied example of intermediate target [1].

What are the signals at the midline, and what are the receptors on the growth cone that control whether axons should cross or should not cross? After crossing once, what mechanism prevents the growth cones from crossing again? Studies in vertebrates and invertebrates [1]–[3] have led to the suggestion that the midline secretes repellent as well as attractant stimuli and that the decision of crossing is regulated by the way growth cones interpret and balance this *concerto* of diverse stimuli. In both vertebrates and insects, commissural axons are initially drawn to the midline by attractant proteins, which include members of the netrin family [1]. However, after crossing, these growth cones lose responsiveness to netrins [4] and become sensitive to repellents made by midline cells, which include Slit proteins [5]–[7].

To gain insights into the molecular mechanism regulating the switch from attraction to repulsion, a large-scale screening was conducted in the beginning of the nineties [8] to identify mutations that affect the pattern of commissural and longitudinal axon pathways in the developing CNS of the *Drosophila* embryo. The screening led to the identification of two key genes: *commissureless* (*comm*) and *roundabout* (*robo*). In *comm* mutant embryos, commissural growth cones initially orient toward the midline but fail to cross it and instead recoil and extend on their own side; as the gene name itself suggests, *commissureless* mutant embryos completely lack commissures [9]. No other mutation bearing a comparable phenotype could be identified in the original or in following screenings [10], [11], thus making *comm* a gene with a unique function. In the absence of *comm* all neurons behave as ipsilateral, whereas overexpression of comm is sufficient to transform an ipsilateral neuron into a contralateral neuron [9], [12], [13]. In fact, *commissureless* is expressed only in contralateral neurons at the moment of midline crossing and it is autonomously required for crossing to happen [12]. At the time of the isolation of the first mutants, it was already proposed that the mechanism by which *comm* might regulate crossing would involve another gene found in the screening, *roundabout* [8]. The *robo* gene encodes for a repulsive receptor for the midline ligand Slit [5], [7], [14]. Of the three Roundabout receptors in *Drosophila*, Robo (the founding member of the family) is primarily responsible for keeping ipsilateral growth cones from crossing and commissural axons from recrossing [15]–[17].

The idea that *comm* regulates crossing by acting on *robo* is based on three different lines of evidence. First, the phenotype in *robo* mutant embryos is qualitatively opposite to *comm* mutant phenotype: in embryos lacking *robo*, many growth cones that would normally extend only on their own side project across the midline, and axons that would normally cross the midline only once, appear to cross and recross multiple times [8]. Second, the double mutants of *comm* and *robo* display a *robo*-like phenotype. Thus, although Comm is essential for axons to cross the midline, in the absence of Robo it is not required at all for crossing [8]. The third important piece of data comes from the analysis of their respective gain-of-function phenotypes: strong overexpression of Robo during embryonic development leads to a *comm* phenocopy. Conversely, overexpression of *comm* leads to a *robo* or *slit* phenocopy and, notably, to a reduction in detectable Robo protein levels [18]. Taken together these observations led to the assumption that Comm controls crossing through regulating Robo. The current view is that Comm acts as an endosomal sorting receptor for Robo [12], [17]. When Comm is absent, Robo is presented on the growth cone where it senses Slit repulsion emanating from the midline, thus preventing crossing or recrossing. Conversely, *comm* expression redirects most Robo to internal compartments, leaving only a lower amount of the protein on the plasma membrane so that the axon can grow unimpeded across the midline.

On a theoretical plan, though, Robo repulsive activity could be modulated through at least two different mechanisms: (I) mere control over protein levels and (II) regulation of the receptor signalling activity. Although the current model focuses entirely on the first process, it is worth noticing that these two regulatory mechanisms are not mutually exclusive. In fact, some pieces of evidence suggest that it is possible that they may well act both at the same time. First, a *comm* mutation has been identified that encodes for a protein that lacks almost the entire cytoplasmic domain, necessary for sorting [12]: this mutant still shows a considerable amount of crossing both in the ventral nerve cord and in the brain (*comm^1^*, [9], [19]) and it is tempting to speculate that the residual crossing activity might be due to a backup mechanism, alternative to sorting. A second striking point comes from the amount of Robo that in physiological conditions escapes sorting degradation. At early stages of neuronal development, when axons first decide whether or not to cross the midline, a high amount of Robo protein is detectable on ipsilateral neuron; yet, contralateral neurons are not completely depleted of Robo on their growth cone and protein on the plasma membrane can be detected using both electron microscopy and regular immunohistochemistry [14], [18]. The presence of Robo on the surface of crossing axons has been interpreted as a way for the growth cone to avoid lingering at the midline [7], [18], [20] but the level of regulation of this phenomenon remains unaddressed. An analogous problem has been described in vertebrates too. It is worth remembering that while the Robo proteins are functionally and molecularly conserved across evolution, no vertebrate *comm* homologues could ever be found: in fact, in vertebrates, regulation of midline crossing is under control of the atypical Robo family member, Rig-1, that acts not by affecting Robo protein expression, but rather by preventing Slit signalling, silencing the Robo protein on the membrane of precrossing axons [20]. Based on these arguments, it was recently suggested that “*flies and vertebrates might both have two mechanisms: one to regulate Robo protein expression (involving Comm in flies and an unknown mechanism in vertebrates) and one to silence low level Robo protein precrossing (involving Rig-1 in vertebrates and an unknown mechanism in flies)*” [20]. Given these premises, it seems indeed reasonable to postulate that in addition to the main role of Comm in sorting Robo, a further finer mechanism could exist silencing the residual repulsive activity of the receptor escaped from the sorting process.

This mechanism, if existing, would be experimentally difficult to unravel, given the major role that sorting indubitably plays. The only way to uncover it would be to inactivate Comm's ability to sort Robo without affecting any other *comm* function. One way this could be achieved would be by generating a mutant form of Robo insensitive to Comm sorting, but still capable of transmitting the repulsive Slit signal in vivo. If regulation of crossing is achieved only by regulating the presence of Robo on the plasma membrane through sorting mechanisms, then the expression of a form of Robo that cannot be sorted would lead to a *comm* phenocopy. Any possible rescue of the expected complete commissureless phenotype could be attributed to a secondary regulatory effect.

In this work I test this hypothesis: taking advantage of a form of Robo insensitive to Comm sorting, I unveil a sorting-independent mechanism of Robo silencing by Comm.

## Results

### Generation of a Comm-insensitive form of Robo

**Figure 1:**
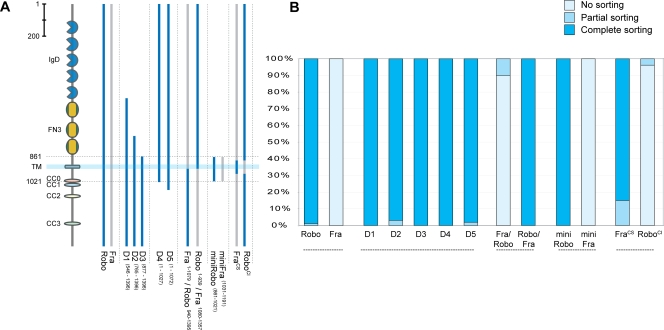
Generation of Robo^SD^–in vitro analysis of Robo deletions and chimerical constructs. (A) Schematic representation of the deletion constructs of Robo and the chimerical constructs between Robo (light blue) and Fra (light gray) used in the COS-7 cell sorting assay. The cartoon on the left part schematizes Robo domain organization. IgD: immunoglobulin-like domain, FN3: fibronectin type 3 domain, TM: transmembrane region, CC0-3: conserved cytoplasmic motifs. All constructs have a N-terminal HA tag and a C-terminal V5 tag (see experimental procedures for details). Robo^SD^ and Fra^CS^ are full length variations of the constructs 18 and 13, described in figure S1. Number in superscript refer to the aa position referred to published sequence (Genbank accession numbers: Robo = gi|2804782, Frazzled = gi|24653090). (B) Quantification of sorting activity as observed in the COS-7 cell assay. Stained cells were analysed and subdivided in three categories as indicated: cells that showed complete localization of Robo and Comm in endosomes and no plasmamembrane staining of Robo were defined as “complete sorting”; cells in which Robo was localized only in the plasmamembrane and Comm only in the endosomes were classified as “no sorting”. Cells that showed Robo distributed at both the plasmamembrane and the endosomes together with Comm were classified as in the category “partial sorting”. Bars show percentage results; at least 50 cells were scored blindly per each experiment.

To map the region(s) of Robo required for its endosomal sorting by Comm I took advantage of a COS cell assay developed in [12] (Figure 1), an in vitro assay thought to reflect the sorting mechanism as it happens in vivo during midline crossing. When *comm* is exogenously expressed in COS cells through transient transfection, the Comm protein localizes in a punctuate intracellular fashion, possibly endosomal and lysosomal structures [12]. Robo, on the other hand, if expressed alone, accumulates mainly at the plasma membrane. In cells that express both *robo* and *comm*, however, the distribution of the Robo protein changes and assumes an intracellular pattern, co-localizing with Comm. Using the relocalization of Robo upon Comm co-expression as a read-out, it was possible to test mutants of the Robo protein for forms that would be insensitive to Comm sorting activity.

Utilizing an unbiased approach, a series of variegated modifications of Robo was constructed, covering the entire protein: three deletions in the extracellular domain (D1, D2, D3 in Figure 1A), two deletions in the intracellular domain (D4, D5 in Figure 1A) and two chimeric proteins between Robo and the netrin receptor Frazzled (Fra) having extracellular and transmembrane domains of one followed by intracellular domain of the other (Robo/Fra and Fra/Robo in Figure 1A). Fra, like Robo, is an immunoglobulin super-family guidance receptor expressed on commissural axons [21]. In contrast to Robo, however, Fra is not targeted for endosomal sorting by Comm [12] and it can therefore be used as negative control. These modified forms of Robo were tested for their sensitivity to Comm in the sorting assay previously described (Figure 1B).

Almost the entire extracellular and cytoplasmic domains of Robo are dispensable for Comm mediated sorting (Figure 1B). The deletion series, in particular, defined a small region (161 aa) between amino acids ^861^FMDP and AEVD^1021^, consisting of the Robo transmembrane domain (23 aa), flanked by 54 extracellular amino acids and 84 intracellular amino acids, that is sorted to endosomes as efficiently as the full length Robo protein (mini-Robo in Figure 1b). The mini-Robo protein localizes in COS cells indistinguishably from full length Robo: it is inserted in plasma membrane when expressed alone, but it is sorted to endosomes when co-expressed with Comm. Importantly, a mini-Fra construct, analogous for size and structure to mini-Robo, is insensitive to Comm and always localizes on the plasma membrane (Figure 1B). Analysis of further modifications of this region (Figure S1) showed the transmembrane domain to be necessary, but not sufficient, for sorting and that almost complete sorting could be obtained using as little as the 83 aa peri-membrane region (aa ^891^HNNG to ESLW^973^: construct 18 in figure S1). This suggests that the peri-membrane region of Robo is sufficient for Comm sorting. Is it also required? To answer this question, a chimeric form of full length Robo was tested in which the region between aa ^891^HNNG and ESLW^973^ was swapped with the analogous region of the Frazzled receptor (analogous to construct 13 in figure S1). As expected, such a modified protein is insensitive to Comm sorting in vitro (from now on it will therefore be referred to as *sorting-defective* Robo or Robo^SD^). Control experiments have also been performed using the netrin receptor Fra or a modified form of Fra, in which the transmembrane and juxtamembrane regions were substituted with the Comm interacting region of Robo (based on construct 18 in figure S1); the latter modified Fra construct (referred to as comm-sensitive Fra or Fra^CS^) is sorted to endosomes in vitro (Figure 1B), while the wild type Fra does not, suggesting indeed that the trans and peri-membrane region of Robo is necessary and sufficient for Robo sorting by Comm.

### Biochemical interaction between Robo and Comm

How does the modification in Robo^SD^ affect sorting? It was previously shown using immunoprecipitation experiments that Robo and Comm form a biochemical complex in vitro [12]; the formation of this complex is likely to be required for sorting, since the interaction is specific for Robo but not for Fra. Interestingly, this association was also shown to require the extracellular and/or transmembrane domains of Robo [12]. Therefore, one obvious possibility is that the binding between Robo and Comm is mediated by the transmembrane and juxtamembrane region of Robo and modifications of this region would disrupt their capability to physically interact and consequentially confer insensitivity to sorting. Immunoprecipitation experiments in COS cells were therefore performed to test this hypothesis.

**Figure 2:**
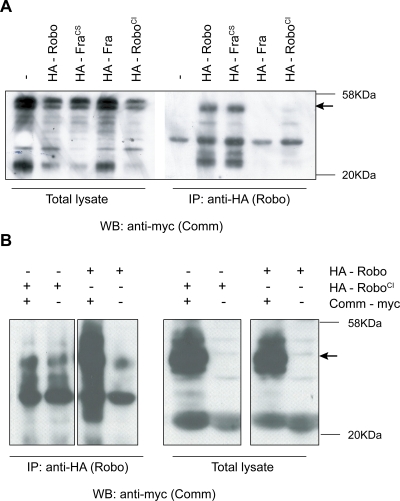
Biochemical interaction between Robo^SD^ and Comm in vitro and in vivo. (A,B) Coimmunoprecipitation of Comm with Robo and Fra^CS^ but not Fra and Robo^SD^ from transfected COS cells (A) and from *Drosophila* embryos (B). In (B) embryos panneuronally expressing a Myc-tagged version of Comm and a HA-tagged version of either Robo or Robo^SD^ were lysed and immunoprecipitated with anti-HA antibodies. The co-immunoprecipitation was assessed by western blot using anti-Myc antibodies. Equal amounts of expression were controlled by western blot on a smaller fraction of lysate. In both panels molecular weight markers are indicated on the right, in kDa.

Lysates from cells expressing both Comm and Robo^SD^ or Comm and Robo were immunoprecipitated with antibodies against the HA tag on Robos and probed on Western blots with anti-myc antibodies to visualize Comm (Figure 2A). As predicted, Comm could easily be detected in the anti-HA precipitates when coexpressed with Robo but not when coexpressed with Robo^SD^, indicating that Robo^SD^ indeed is not able to associate with Comm. The control constructs (an HA tagged version of the wild type Fra or of the Fra^CS^) also confirmed the results obtained with the sorting assay, with Fra^CS^, but not Fra, being able to physically associate with Comm (Figure 2A).

### Robo^SD^ is insensitive to Comm degradation in vitro

Previous in vivo data showed that protein levels of Robo and Comm in the embryo are inversely correlated: using transgenic constructs it was shown that the overall levels of Robo are dramatically decreased wherever an increased Comm expression coincides [9], [17], [18]. This decline in the level of Robo protein is thought to be a consequence of active protein degradation as the ultimate step of Comm sorting into the endosomal compartment.

**Figure 3:**
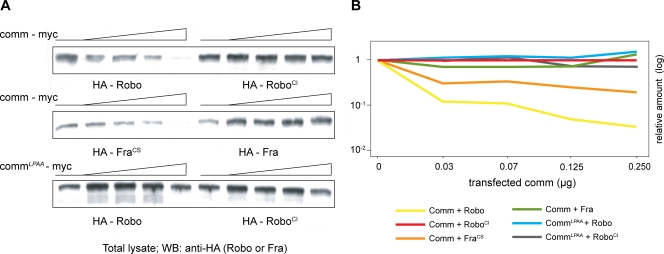
Robo^SD^ is insensitive to Comm degradation in vitro. (A) Western Blot analysis showing an inverse correlation between total levels of detectable Robo and growing amount of Comm protein in COS7 cells. Cells were transfected with 0, 0.03, 0.06, 0.120 and 0.250 micrograms of Comm-myc (upper two panels) or myc tagged Comm^LPAA^ (lower panel) plasmidic DNA and 0.5 micrograms of HA-tagged Robo, Robo^SD^, Frazzled or Frazzled^CS^. (B) Quantification of signal intensity of (A).

In the next experiment two points were then addressed: (i) whether the protein degradation that was observed in vivo upon Comm overexpression could be reproduced by analogous experiments in vitro and, if yes, (ii) whether Robo^SD^ would be insensitive to this degradation as predicted. To answer these questions an in vitro assay was established, based on the effect of the simultaneous expression of Comm and Robo in COS cells (Figure 3). In a comparative experiment, COS cells were transfected using a constant amount of Robo and five serial concentrations of Comm (from 0 to 0.250 micrograms); after 48 hours from the transfection, cells were harvested, extracted and normalized for their total protein content, and total levels of Robo were analyzed by western blot using a monoclonal antibody directed against the HA tag. As predicted, the result showed a clear inverse correlation between the amount of detectable Robo protein and levels of transfected Comm: the higher the amount of transfected *comm*, the lower the levels of detectable Robo. As control experiment, a modified form of Comm was used in which the conserved endosomal sorting signal 229LPSY was partially mutated; the unmodified Fra receptor was also used as control. Point mutations in the LPSY motif were previously shown to severely impair Comm function in vivo, just as they prevent endosomal sorting in vitro (L229A, P230A as described in [12], [13]). As anticipated, neither a variation in Robo protein levels was detectable upon coexpression of this modified form of Comm, nor a reduction of Fra upon Comm expression. This suggests that, similarly to what happens in vivo, in vitro sorting of Robo by Comm into the endosomal compartment eventually results in a reduction of detectable Robo protein levels. What about Robo^SD^? When the same experiment was repeated using Robo^SD^ instead of Robo, no significant diminution in protein levels was observed. Robo^SD^ levels are not affected by Comm expression. This result indicates that the Comm-binding region of Robo is not only sufficient to mediate the biochemical interaction (as shown in Figure 1 and 2) but also to lead to successful protein degradation (Figure 3).

In this set of experiments Robo^SD^ proved to be unable to form a biochemical complex with Comm, unable to undergo sorting into the endosomal compartment and, as a consequence, is unable to be a target of Comm-induced degradation.

### Robo^SD^ is insensitive to Comm action in vivo

**Figure 4:**
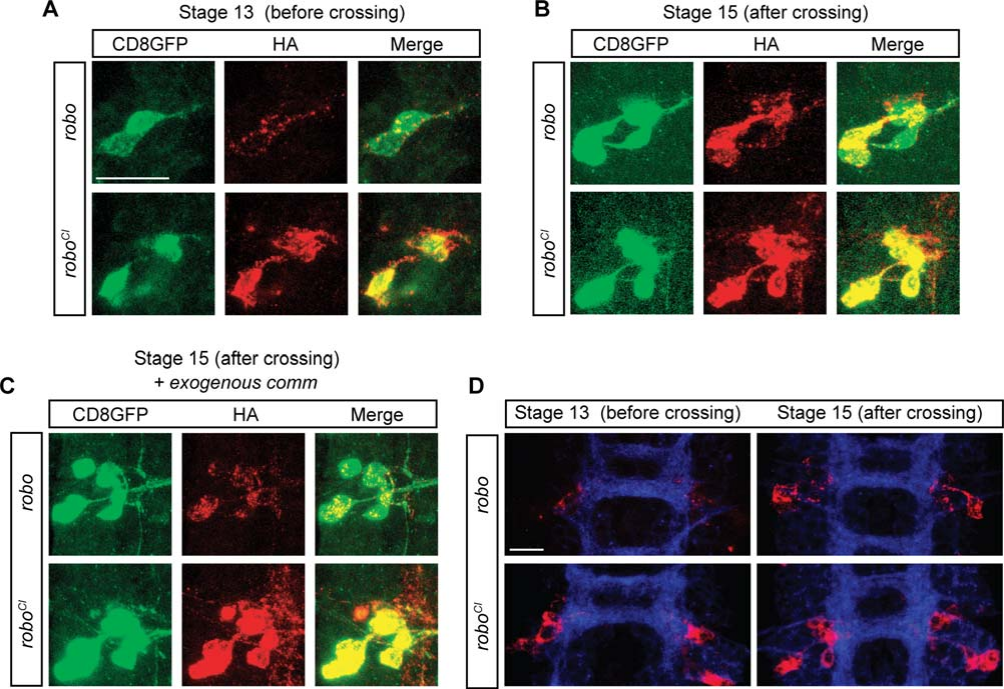
Robo^SD^ is insensitive to Comm sorting in vivo. (A–B) Confocal images of single cells expressing the fluorescent marker CD8GFP (green) and an HA-tagged version of either Robo or Robo^SD^ (red). Panel (A) shows the soma of poxn-GAL4 expressing cells at stage 13 of embryonic development, namely while their growth cone is crossing the midline; panel (B) shows analogous cells at stage 15, after the crossing is complete. Notice that while there is no difference between Robo and Robo^SD^ localization at stage 15 (B), a clear difference is observable at stage 13 (A). (C) Same experiment as in (B) but with ectopic expression of a uas-Comm transgene. (D) Lower magnification images of one segment of the CNS of UAS-HA-Robo (upper two) and UAS-HA-Robo^SD^ (lower two) expressing embryos, before crossing (leftmost two) and after crossing (rightmost two). Images in panels A–C are acquired using 100× magnification; images in panel D are acquired using 63× magnification. White size-bar indicates 10 µm.

Having established that Robo^SD^ is not sorted by Comm in COS cells, it became fundamental to confirm its insensitivity to Comm in neurons, in vivo. To test this, an assay conceptually analogous to the one already adopted in vitro was established (Figure 4). Overexpression of Comm during development from an uas-Comm transgene leads to two apparently related consequences: a substantial reduction in Robo protein levels as detected by immunostaining and a lack-of-repulsion phenotype, reminiscent of a *robo* loss-of-function phenotype [18]. Both the extent of protein reduction and the intensity of the phenotype are dosage sensitive, being proportional to the amount of *comm* expressed: for instance, a strong overexpression of uas-Comm by means of two transgenes simultaneously expressed, leads to an almost complete depletion of Robo protein and to a CNS phenotype resembling the *slit* loss-of-function, in which all axons collapse into the midline [15], [16], [18]. These phenotypes are thought to be a consequence of downregulation of Robo receptor by virtue of a sorting mechanism similar to the one characterized in vitro. In fact, Comm accumulates in neurons in a vesicular pattern, a localization that resembles the one observed in COS cells [9], [22] and co-expression of Comm and Robo in neurons results in a relocalization of Robo from the plasma membrane to a vesicular compartment, as it happens in vitro [12]. Given the assumption that the sorting observed in vivo is mechanistically analogous to the one characterized in vitro, one could predict that the behaviour of Robo^SD^ in the embryonic CNS would not be dissimilar from the one just characterized in the COS cell assays.

To address this point, I used the poxn-GAL4 driver to express a wild type form of Robo or its Comm-insensitive counterpart in an easily identifiable set of cells in the CNS of the *Drosophila* embryo. The poxn-GAL4 is expressed starting from stage 12/13 of embryonic development in contralaterally projecting neurons allowing thus to compare the distribution of Robo or Robo^SD^ before and after the midline crossing, namely during different states of *comm* expression [17]. To be able to identify the neurons and their developmental stage, as well as being able to delineate their shapes and contours, poxn-neurons co-expressed a protein fusion between the transmembrane domain of mCD8 and GFP (uas-CD8GFP; left green panels on Figure 4). To clearly identify the developmental stage, the entire CNS was also stained using an anti-HRP antibody (Figure 4D). At stage 13, the growth cones of the most medial cluster of poxn neurons are approaching or just crossing the midline; visualization of a uas-Robo at this time revealed a punctate pattern in the soma of the neurons, very similar to the vesicular pattern observed in COS cells when *comm* is co-expressed (Figure 4A and 4D). After crossing, (stage 15) Robo localization changed dramatically, increasing of intensity (Figure 4D and B) and appearing as a clear plasma membrane staining co-localizing with the CD8GFP protein (Figure 4B). An analogous change was originally described in [17] and it is likely to reflect the intrinsic change in *comm* expression and therefore to constitute the read-out of Robo sorting in vivo. What happened then to Robo^SD^? Contrarily to Robo, Robo^SD^ localization was quantitatively and qualitatively constant in time: prior and after crossing, Robo^SD^ localized on the plasma membrane of the neurons together with mCD8GFP (Figure 4A, B, D). In order to strengthen this observation, and to confirm the role of comm behind the change in Robo localization observed in Figure 4AB, a similar experiment was performed, this time with a simultaneous expression of uas-Comm (Figure 4C). In these conditions, Comm would be immediately and constitutively expressed in the poxn-gal4 neurons and this should result in a constant sorting and degradation of Robo protein, both before and after crossing; conversely, Robo^SD^ should not be affected by the presence of Comm. This was indeed the case (compare red panels of Figure 4B and 4C).

To further test the insensitivity of Robo^SD^ to Comm in vivo, embryos expressing panneuronally both Comm and Robo^SD^ or Comm and Robo were lysed and immunoprecipitated with anti-myc antibody directed against the myc-tagged Comm; western blots were then probed using anti-HA antibody to detect Robo protein (Figure 2B). No interaction was observed when Comm was coexpressed with Robo^SD^, once again indicating that the ability of Robo^SD^ to associate with Comm is deeply affected, in vivo as in vitro.

### Targeted insertion of Robo^SD^ into the *robo* locus

Having established that Robo^SD^ is indeed insensitive to Comm sorting in vivo, I could proceed addressing the original question and analyse the effects of Robo^SD^ expression during development of the nervous system. According to the current model, Robo is regulated only by endosomal sorting. If this is really the case, introducing in the embryo a new form of Robo that cannot undergo sorting would result in a Robo gain-of-function phenotype (or *commissureless* phenocopy), as this modified protein should be constitutively active. Any residual crossing should be a consequence of an additional form of regulation, sorting independent.

For the correct interpretation of the experiment, it was paramount to exclude any confounding factor in the regulation of Robo. For this reason, the *robo* gene was targeted with homologous recombination to create new sorting defective alleles, so that the modified Robo proteins could be expressed in a manner otherwise identical to their wild type counterpart. Leaving the regulatory region of the gene untouched (as in fact most of the coding sequence) could allow for the reproduction of the exact temporal and spatial expression of Robo, as well as for maintenance of the expression levels of the protein as similar as possible to wild type. Three new alleles of *robo* were generated: in all of them exons 15 and 16 (encoding for the 83 amino acids spanning the transmembrane and juxtamembrane domain; see figure S2 for details on the sequence) were replaced with one longer exon. In Robo^SD-Fra^ the sequence was substituted with the analogous region of the Frazzled gene; in Robo^SD-CD8^ with the transmembrane and juxtamembrane part of the murine CD8 receptor. Robo^CS^ was the control modification, in which only the gene structure changed but the protein sequence remained unaltered (Figure S2). According to the model, Robo has to be efficiently sorted for degradation in order for crossing to happen. Therefore, even a slight amount of Robo^SD^ protein could possibly lead to a *comm* phenocopy and be dominantly lethal. To overcome this possibility, three more alleles of Robo^SD^ were engineered in a conditional way, in which the modifications described above were preceded by a loxP flanked cassette inserted in the 14–15 intron and containing (in order from 5′ to 3′): a short 15–16 exon encoding four copies of a myc tag and the endoplasmic reticulum retention sequence (KDEL [23]), a stop codon and a SV40 poly-A tail. The KDEL element was used to prevent any potential partial peptide translated from the residual 5′ *robo* gene from being secreted. The loxP elements were used to allow Cre mediated excision of the cassette in a cell autonomous manner (Figure S3).

Targeting of all six constructs was achieved as described in [24]. Out of ∼400.000 flies screened, 70 were found positive. The precise insertion of each construct was confirmed by PCR amplification and Southern Blot analysis (Figure S2). Two independently obtained lines per genotype were used for the phenotypical analysis.

### Expression of Robo^SD^ in vivo

**Figure 5:**
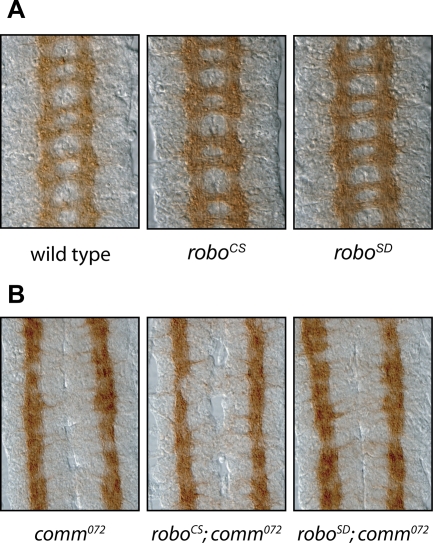
Wild type CNS Phenotype of Robo^SD^ targeted alleles. (A) Embryonic CNS phenotype of targeted alleles as reveled by BP102 staining. All modifications show a wild type looking CNS. (B) BP102 staining of embryonic CNS of Robo^SD^ targeted alleles in a *comm* mutant background.

To much of a surprise, Robo^SD^ flies were viable and fertile and, more importantly, CNS of Robo^SD^ homozygous embryos did not show any phenotypical abnormality (Figure 5A and figure S4). Embryos homozygous for Robo^SD-Fra^ and Robo^SD-CD8^ were collected at different stages of development and their CNS was stained and dissected; in all experiments Robo^CS^ and Canton-S flies was used as control genotypes. No abnormal phenotype was observed at any stage of embryonic development of the CNS, from early 12, when axon outgrowth begins, to late 16, when embryos are about to hatch (Figure S4A). Embryos were stained using both pan-neuronal antibodies (e.g.: BP102 in figure 5 or anti-HRP in figure S4A) or subset specific antibodies (such as ID4, figure S4A). Despite the thorough analysis and the blind scoring, it was not possible to find any difference between the CNS of Robo^SD^ targeted embryos and the control alleles of Robo. In particular, commissures formation and distribution of the lateral fascicles (the two main processes regulated by Robo receptors) occurred just normally in all examined embryos. Notably, localization of the Robo^SD^ protein also appeared identical to Robo, both at early stages and late stages of development (figure S4B). In particular, Robo^SD^ was detectable on the longitudinal fascicles but not on the commissural tract, suggesting that localization of Robo does not depend on *comm* action, as previously hypothesized [7].

**Figure 6:**
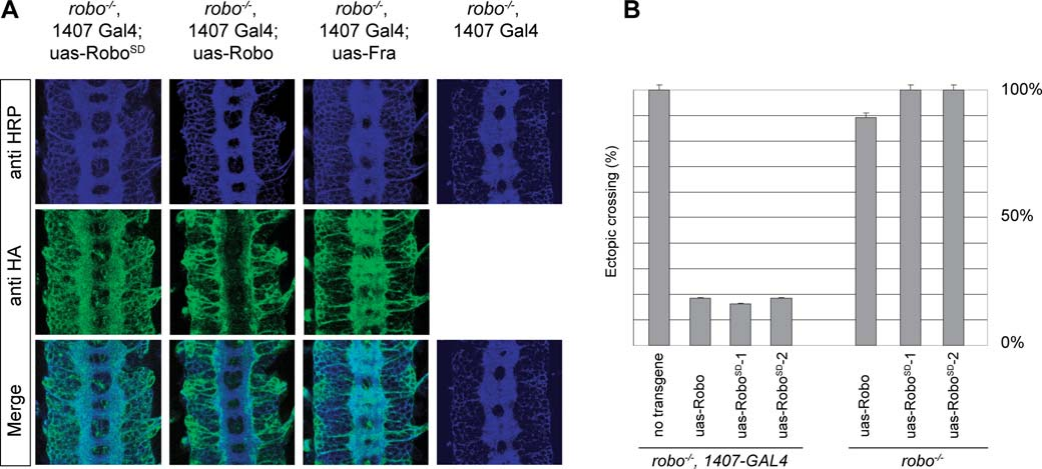
Rescue of a *robo* mutant with Robo^SD^ transgenes. (A) Expression of Robo or Robo^SD^ transgenes in a *robo^1^* mutant embryo using the UAS-GAL4 system completely rescues the commissural phenotype while expression of Frazzled does not. (B) Quantification of above. Embryos of the indicated genotype were stained with ID4 (anti-Fas2) antibody labeling the longitudinal fascicles and dissected to score the percentage of ectopically crossing fascicles. Two independent insertions of a Robo^SD^ transgene were used and all showed levels of rescue comparable to the uas-robo control.

Given the surprising result of this experiment, it was paramount to exclude possible trivial artefacts due to the way the experiment was designed. One first possibility could have been that the modification introduced to generate the Robo^SD^ allele would somehow confer less repulsive force to the receptor activity. To test this hypothesis, two different experiments were conducted. The aim of the first experiment was to analyse the phenotype of removal of *comm* in a Robo^SD^ background: assuming that the mechanistical explanation of the *commissureless* phenotype is the excess of Robo repulsive signalling, if the repulsive activity of Robo^SD^ was indeed affected by the protein modification, one would have observed again a more *wild type* looking phenotype or at least a less severe *commissureless* phenotype. Once again, no difference was detectable and in all cases removal of *comm* led to complete absence of commissures (Figure 5B). In a second set of experiments, the GAL4-UAS system was used to drive expression of transgenic forms of wild type Robo or Robo^SD^ in the background of a *robo* mutant, in the attempt to quantify and compare their ability to rescue a *robo* phenotype (Figure 6). In absence of a rescue construct or with the uas-Fra transgene all segments showed a *robo* phenotype when analysed with the mAb ID4; both expression of uas-Robo or uas-Robo^SD^ could revert the phenotype almost completely, with ectopic crossing observable in only 20% of segments in both cases (Figure 6A and 6B). These results indicated that the absence of a *commissureless* phenotype upon Robo^SD^ expression could not be attributed to an intrinsic defect of the modified Robo receptor but rather to a novel mechanism of regulation.

### A sorting independent mechanism of Robo signaling downregulation

Given that Robo^SD^ has proved to be insensitive to Comm-mediated sorting both in vitro and in vivo, and given that the Robo^SD^ receptor does not show any sign of impaired signalling capabilities in a rescue assay in vivo, it is tempting to conclude that the correct midline crossing observed in the Robo^SD^ flies is due to a secondary regulatory mechanism, acting not on the protein levels but rather on a different aspect of Slit signal propagation. If this is the case, it should be possible to envision a scenario in which silencing of Robo can be achieved independently of its degradation. In other words, an embryonic CNS that would display at the same time a slit phenocopy and high levels of Robo protein.

**Figure 7:**
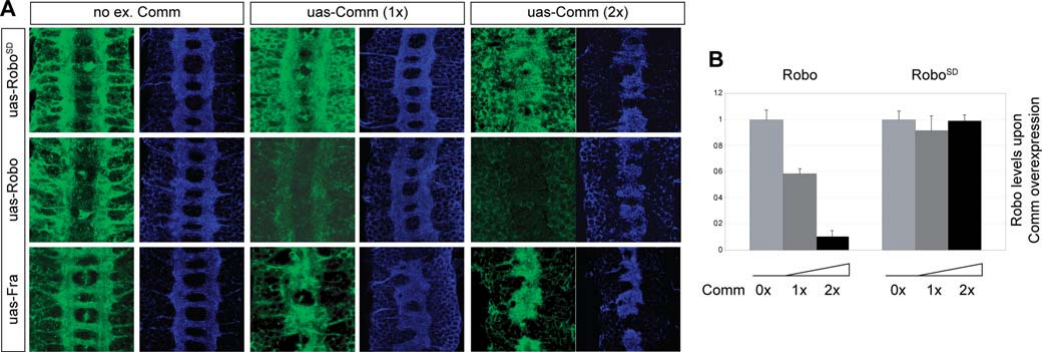
Overexpression of Comm induces a Slit phenocopy without reducing the amount of Robo^SD^. (A) CNS phenotype of embryos expressing none, one, or two copies of a Comm transgene using the GAL4-UAS system. Detectable levels of ectopically expressed HA-Robo drop proportionally to the amount of Comm expressed while HA-Fra, and HA-Robo^SD^ do not show any reduction. In all cases overexpression of Comm induces a Slit phenocopy. anti-HA staining (Robo, Robo^SD^ or Fra) is shown in green. anti-HRP staining (pan-neuronal marker) is shown in blue. (B) Quantification of detected protein levels of the experiment in (A).

To conclusively test this possibility, the pan-neuronal driver 1407-GAL4 was adopted to simultaneously express an HA tagged version of uas-Fra, uas-Robo or uas-Robo^SD^ and an increasing amount of uas-Comm (Figure 7). Embryonic phenotypes were then analyzed using the neuronal marker anti-HRP and the relative amounts of Fra, Robo or Robo^SD^ proteins were assessed using anti-HA antibodies. In the absence of the UAS-Comm transgene, Robo and Robo^SD^ are expressed at similar levels (Figures 7A). As expected, simultaneous expression of uas-*comm* resulted in a strong reduction of Robo levels but not of Robo^SD^ or Fra. Increasing the amount of overexpressed comm, using two copies of the uas-*comm* transgene led to an even more striking difference, where Robo levels became undetectable while Robo^SD^ levels stayed unchanged. Importantly, in all cases the phenotype reflected only the amount of *comm* expressed and not the amount of residual Robo or Robo^SD^ protein: in particular, overexpression of two copies of the uas-comm transgenes led to a complete *slit* phenocopy, despite the high levels of Robo^SD^ protein detected (Figures 7A and 7B).

In other words, overexpression of *comm* in the developing nervous system can lead to a *slit* phenocopy, not only by removal of the Robo protein through sorting but also through another mechanism of unknown nature that most likely involves inhibition of repulsive signalling.

## Discussion

In the Drosophila CNS, some axons but not others cross the midline. The decision to cross or not to cross the midline is controlled by Comm. Expression of Comm in *Drosophila* embryos leads to a decrease in the amount of detectable Robo protein [18], through a process that involves sorting of the Robo receptor in the lysosomal compartment [12], a phenomenon that has been assumed to be the underlying mechanism for control of midline crossing. Nevertheless, not all Robo is successfully sorted for degradation and a small amount of the receptor is still localized on the membrane of crossing axons [14], [18]. Why so? A possible explanation, already advanced in [18], is that “*this system has evolved to prevent lingering at the intermediate target, that is, to make sure that axons that enter the midline actually cross and leave it. If commissural growth cones did not express Robo, they might be tempted to linger at the midline.*” If this is the case, how is this mechanism tuned? A control of Robo signalling activity independent from sorting has to exist, analogously to what was observed in vertebrates [7], [20]. In this work I concentrated on this problem and revealed a second level of regulation in controlling Robo signalling.

To address the problem, I decided to interfere with Robo sorting, abolishing it. In this way, every observed residual effect of regulation on a sorting-insensitive Robo could then be attributed to a secondary mechanism of control.

Generation of a sorting insensitive Robo was achieved through an in vitro assay and an unbiased approach that led to the mapping of the peri-membrane domain of Robo as the only Comm-interacting domains. In particular, the transmembrane domain itself seemed to be necessary for sorting to happen but not sufficient (figure S1A,B). Notably, a small Fra-Robo chimeric construct carrying only the transmembrane domain of Robo (construct 16 in figure S1) still showed some degree of interaction with Comm (figure S1C) but hardly any sorting, suggesting that either the interaction is not stable enough for sorting to happen or that another portion of the peri-membrane region is required. It is important to notice that complementary experiments done on the Comm protein led to the analogous conclusion, namely that the association between Robo and Comm requires an intact transmembrane and juxtamembrane region of Comm [17], [22], [25]. Compatibly with this observation, only two stretches in the primary sequence of Comm were shown to be evolutionarily conserved: the so called LPSY sorting motif and the transmembrane and juxtamembrane region [12], [22]. Here, I showed that the Robo peri-membrane region is important for the formation of a biochemical complex with Comm both in vitro and in vivo (Figure 1 and 2) and fundamental for correct sorting (and subsequent protein degradation) to happen, both in vitro and in vivo (Figure 3 and 4). It is not yet clear what the molecular mechanism is that regulates the interaction between the two proteins. The fact that a successful sorting could be reproduced in *Drosophila* S2 cells [22], [26] and in mammalian COS cells [12] suggests that the interaction between Robo and Comm could be direct, without need for a bridging partner.

The modification of the Robo peri-membrane domain does not affect its repulsive activity or its ability to localize at the plasmamembrane, yet embryos and flies expressing Robo^SD^ are completely wild type suggesting that not only a secondary mechanism exists but also that it is completely redundant to Robo sorting itself. On this matter, the most informative and compelling observation comes the overexpression of Comm in the Robo^SD^ background (Figure 7). In that case, Comm was able to induce a perfect phenocopy of the slit mutant, despite the detectable levels of Robo^SD^ did not change. This result is strong evidence that downregulation of repulsion does not act only by downregulation of Robo protein levels, but most likely also by downregulating Robo signalling, through some other unknown mechanism.

Finally, it is worth noticing that localization of Robo^SD^ is not different from the localization of its wild type counterpart: Robo^SD^ is detectable along the longitudinal fascicles but not on the commissural tracts (figure S4B). This localization pattern is common to all three Robo receptors [15], [16], but while clearance from commissures of Robo2 and Robo3 was already shown to be independent of comm activity [15], localization of Robo was still assumed to be under control of Comm. The data presented in this paper show for the first time that this is not true. What is then the molecular mechanism behind commissural clearance of Robos? Two alternative models were already proposed in the past [7]: Robo clearance could happen as a consequence of a passive mechanism (for instance: a compartmentalization in the plasmamembrane) or an active mechanism. A series of still unpublished experiments suggest this latter to be the case and that clearance of Robo from commisures is a consequence of ligand induced endocytosis of the receptor (GFG, manuscript in preparation).

### Two different mechanisms to control the same event

Regulation of midline crossing is arguably one of the best studied and understood process of axon guidance. Numerous animal models have been exploited in the last few decades to unveil the molecular mechanism behind this relatively simple choice, from the grasshopper ventral nerve cord, to *Drosophila*, to the vertebrate optic chiasm, cerebellum and floor plate. From a galore of fascinating experiments we learned that the same result (to cross or not to cross the midline) could be achieved using a number of different genes through different regulatory mechanisms depending upon the neurons and the context (ephrins, plexins, robos). Even within the best understood model of regulation of midline crossing, the Slit/Robo/Comm system, there is a consistent number of pleiomorphisms in the way the regulation is achieved: in the genome of many insects at least three Robo receptors are present; at least two are known to contribute to crossing in *Drosophila.* Also, there are three *comm* genes in *D. melanogaster* and other insects. It should not be a surprise if such an important step of regulation in the life of a neuron (such as midline crossing) is under control of multiple mechanisms.

In vertebrates, regulation of Robo is under control of the Robo family member Rig-1 through a mechanism that is different from sorting. Yet, in vertebrates, like flies, Robo protein is dramatically upregulated after midline crossing, independently of the presence of Rig-1. It has been proposed that this regulation may use a mechanism that is similar to the one observed in flies, perhaps through a still to be discovered Comm-like protein [20]. Therefore it is not completely surprising to see that in flies, Robo repulsive signalling is under control of at least two different mechanisms.

Importantly, a sorting-independent mechanism could play two roles: evolutionarily, as a backup system of Comm silencing, to be used when sorting fails for unpredictable reasons; physiologically, to silence the Robo receptor that remains on the growth cone during crossing. There are arguments to think that a small amount of Robo on the growth cone is not just an accident: sudden activation of that little Robo present on the growing tip of the axon immediately after reaching the midline could be an efficient way for the neuron to overcome stalling and provide a quick switch from attraction to repulsion. Nevertheless, a system should exist to silence repulsion until the moment is right, namely until the midline is reached.

### Molecular mechanism of sorting independent silencing

It is difficult to say, at this point, what the molecular mechanisms underpinning the silencing described in this work are: we know for sure that the *comm* gene is required for midline crossing to happen, since *comm* mutants have an unmistakable commissureless phenotype. Thus, it is safe to conclude that the sorting-independent regulation of Robo is under the control of Comm, although not in a direct way since it does not seem to require the formation of a complex (see figure 2). Comm is indubitably a versatile molecule; other than acting as a sorting receptor for all Robo proteins, it has been shown to play a crucial role in the formation of synapses at the neuromuscular junction, through a mechanism that does not involve sorting but instead endocytosis [27], [28]. Sorting independent silencing could be initiated by Comm and could then be mediated by other molecules. Good candidates for this role could even be the other Robo receptors, exactly as it happens in mammals. In 2000 Simpsons et al. [16] showed that Robo2 overexpression in the CNS could induce a completely unexpected phenotype: while higher levels of Robo2 overexpression would lead to a commissureless phenocopy, a more contained overexpression would lead to a qualitatively opposite phenotype in which axons seem to be attracted towards the midline rather than repelled, eventually resulting in phenotype resembling the *robo* mutant. Based on this observation in that paper the authors suggested that Robo2 can interfere with or decrease the output of Robo signalling. The involvement of a completely different molecular mechanism would explain why alleles of *comm* that completely lack the LSPY motif necessary for sorting are still able to induce crossing [9]. Another possible explanation is that Robo may not be the only target of Comm sorting; it is possible that Comm is acting as a sorting receptor both for Robo and for one or more molecules downstream of the Robo pathway. This explanation would be compatible with the observation that a *robo*;*comm* double mutant looks like *robo* and, economically, it would not require to introduce a completely new molecular mechanism but simply a new target. A conspicuous number of cytoplasmic proteins [29]–[33] and transmembrane proteins [34]–[38] have been genetically or biochemically involved with Robo signalling. Mutants in most of these genes have been shown to induce phenotypes which are similar or sometimes identical to *robo* mutants. It is therefore possible to postulate that *comm* may act on any of these molecules alone or in combination and that it may do this through a mechanism that involves the sorting machinery.

## Materials and Methods

### Generation of Robo^SD^ Mutants

Gene targeting by homologous recombination was performed essentially as described by [39] and illustrated in details in figure S1. Targeted lines were selected by mobilizing and linearizing the original donor using hsFLP and hsI-SceI and crossing these virgin to eyFLP [40] males so that reintegration can be detected in the progeny by the stable expression of the *white*+ reporter. Between two and ten independent lines were obtained from each of the original donor elements. Southern Blot and PCR analysis were used for genomic confirmation of the proper targeting.

### Immunohistochemistry

Immunofluorescence staining of fixed Drosophila embryos was performed as described [15], using mAb Bp102 (1∶750), anti Fas-II mAb ID-4 (1∶1000), anti-HA mAb 3F10 (1∶1000, Roche Diagnostics), mAb anti-Robo (1∶100), anti-B-galactosidases (1∶1000, Promega) and Cy5-conjugated sheep anti-HRP (1∶500, Jackson Immunoresearch) primary antibodies, with Alexa Fluor-568 and Alexa-488 conjugated secondary antibodies (1∶1000, Molecular Probes). When needed, embryos were genotyped using anti-β-galactosidase staining to identify embryos carrying lacZ expressing balancer chromosomes. Selected embryos were dissected, mounted in Vectashield mounting medium (Vector Labs) and confocal images acquired on a Zeiss LSM 510 microscope.

### Cos cells transfection and Immunofluerescence staining

In vitro analysis of Robo sorting in COS cells was conducted as described in [12]. Cells were counted and classified in a blind condition. Due to nature of the experiment 20–30% of transfected cells shows mislocalization of Comm on the plasma membrane; to our purposes only cells showing a correct localization of Comm were used for scoring the localization of Robo.

### Plasmids

All constructs of robo (including wild type, full-length modifications and mini-robo modifications) used for transfection in COS cells were prepared in the pUB6/V5-HisA vector (Invitrogen) and all carried three copies of the HA tag in their N-terminal portion and a copy of the V5 tag followed by an His-Tag in the C-terminal portion. For deletions of Robo standard PCR-based cloning procedures were used while for generation of the chimerical constructs we used the overlap extension PCR method. Primers used for construction of all plasmids and PCR details are in figure S2. In all cases the integrity of each plasmid was confirmed by sequence analysis. Each of these predicted proteins contains the Wingless signal sequence followed by three HA epitope tags at their N-terminal and theV5 epitope and H6 tags at their C-terminal. comm-myc and derivatives were already described in [12]. These wild-type and mutant Robo inserts were then subcloned into pUAST to generate UAS-Robo transgenes.

### Immunoprecipitation

In vitro co-immunoprecipitation experiments were performed as in [12]. In vivo co-immunoprecipitation experiments were performed as in [41]. Briefly, 0- to 15-hour old embryos were collected and dechorionated with bleach for 3 minutes. 0.1 ml of embryo were homogenized with 0.2 ml of lysis buffer (50 mM Tris-HCl, pH 7.5, 150 mM NaCl, 1% NP40, 0.5% sodium deoxycholate, 1 mM DTT, 1 mM PMSF). Lysate was centrifuged at 13,000 g at 4°C for 10 minutes. The supernatant was pre-absorbed with 50 µl protein A-agarose beads (Amersham Corp) at 4°C for 30 minutes with gentle agitation to eliminate non-specific binding of the proteins to the beads. Protein A-agarose beads were separated from the lysate by centrifugation for 1 minute at 13,000 g at 4°C. Anti-HA (1∶1000) or anti-myc (1∶250) was incubated with the lysate for 1 hour at 4°C with gentle agitation. 25 µl of protein A-agarose beads were subsequently added to each lysate/antibody mixture and incubated over night at 4°C with gentle agitation. The immune complex was pelleted by centrifugation at 3,000 g at 4°C for 1 minute. The complex was washed 2 times with low stringency (50 mM Tris-HCl, pH 7.5, 500 mM NaCl, 0.1% NP40) and high stringency (50 mM Tris-HCl, pH 7.5, 0.1% NP40) buffers. The proteins were eluted by boiling the beads with 20 µl SDS-PAGE buffer for 10 minutes.

### Western Blot

Eluted proteins were separated on 8% SDS-PAGE gel and electroblotted to PDF membrane (Bio-Rad). The blots were blocked with PBS containing 5% non fat milk and 0.3% Tween 20 (Sigma). The membranes were incubated with anti-HA and/or anti-myc antibodies at 1∶5000 dilution followed by peroxidase-conjugated antibody at 1∶10000 dilution. After washing with buffer containing PBS and Tween 20, protein bands were visualized with ECL detection kit (Amersham Corp).

## Supporting Information

Figure S1 **Generation of Robo^SD^–in vitro analysis of miniRobo chimerical constructs.** (A) Schematic representation of the 24 chimerical constructs between the mini-Robo (light blue) and mini-Frazzled (light gray) proteins. The total length of the constructs varies between 255 aa and 268 aa. All constructs have a N-terminal HA tag and a C-terminal V5 tag. Numbers on the left side of each contruct indicate the aa position at which the chimeric ligation occurred. (B) Quantification of sorting activity as observed in the COS-7 cell assay for the 24 constructs listed in (A). (C) Co-immunoprecipitation of Comm with the chimerical constructs 13–18, mini-Robo and mini-Fra. Molecular weight markers are indicated on the right, in kDa.
(3.71 MB TIF)

Figure S2 **Targeted constructs and molecular validation of recombinant flies.** (A–C) Schematics of *robo* gene structure in wild type and in its modified forms. Blue boxes indicate exons not modified by the gene targeting. Orange boxes indicate modified exons 15 and 16 encoding for the trans- and juxta-membrane domains. Gray boxes indicated untranslated regions. Exons 15 and 16 encode for the peri-membrane region (from ^858^ISLF to NCRK^1032^). In the homologous recombinants exons 15–16 have been replaced by one single exon (red asterisk). The newly created gene lacks therefore the intron 15–16 and this allows to easily screen a conspicuous number of lines for proper insertion with PCR amplification using primers sitting on the edges of the two exons (green arrows, see also F). In the conditional modification of Robo (*robomyc-loxP*, C) a 1 kb sequence precedes the modified exon and prevents the protein from being expressed (see text for details on the sequence). After excision of the loxP cassette, the genomic sequence does not differ from the one of the constitutive alleles in B. (D) Sequences of the modified proteins in the region translated by the new artifical exon 15+16. Blue indicates the original Robo sequence; red indicates the original Fra or CD8 sequences; the predicted transmembrane domains are underlined. In Robo^SD-Fra^ the sequence of Robo comprised between aa ^891^HNNG and ESLW^973^ is replaced by the sequence of Fra from aa ^1061^QEPD to KGLH^1143^. In Robo^CD8-Fra^ the sequence of Robo comprised between aa ^881^GRHE and LWID^975^ is replaced by the sequence of mouse CD8 comprised between aa ^151^STTT and RSRK^224^ plus a 26 aa linker sequence derived from a mCD8-GFP fusion protein. (Robo = gi 2804782, Frazzled = gi 24653090, mCD8 = gi 1049227). Robo^myc-loxP^ is the protein that is produced in the conditional alleles, ending at aa ISLF^861^ and terminating with four copies of a myc tag and a ER retention sequence (KDEL [23]) to avoid secretion of a potential dominant negative form. (E) Southern Blot analysis showing the difference between a correctly targeted line and a control line. (F) PCR amplification of exons 15 and 16 in some of the modified Robo lines (p283-p56) and in a control line (p279). Higher bands correspond to longer fragments, containing the intron. P283 and p52 are alleles of RoboSD-Fra that did not successfully resolve the duplication after I-CreI excision. p53 is one of the allele of RoboSD-Fra used in this work. P56 is an allele of Robo^CS^.
(2.74 MB TIF)

Figure S3 **Schematics showing the structure of a construct used to generate a Robo^SD^ allele by homologous recombination.** The flox-able version of Robo^SD-Fra^ as an example of a targeting construct used in this work. (A) Successfully transformed flies have the entire targeting construct randomly inserted in the genome. (B) These flies are crossed to ey-FLP, I-SceI expressing flies to excise the targeting construct and start the process of homologous recombination. (C) If the targeting by homologous recombination is successful, the targeted region is modified with a partial duplication of the gene and the inclusion of the modified form. (D) Crossing this targeted flies to I-CreI expressing flies will excise a linear fragment of DNA and resolve the duplication removing also the *white* mini-gene used as marker for transgenesis. (E) In case of the conditional alleles, the Cre enzyme is used to remove a loxP cassette.
(1.03 MB TIF)

Figure S4 **Phenotypic analysis of robo^SD^ embryonic CNS.** (A) Representative pictures of a wild type CNS at all stages of development in *roboSD* embryos (left three columns) and *roboCS* (wild type control) stage 15 embryos (rightmost column). Embryos were stained using anti-HRP antibody (upper row, gray), directed against a pan-neuronal marker and labeling the entire nervous system and anti FasII antibody (ID4, middle row, red) labeling three longitudinal fascicles running along the longitudinal line. Bottom row provides a merge of both channels. (B) Distribution of the Robo^SD^ protein during development (left three columns) and Robo^CS^ (rightmost column) at stage 15. Distribution of Robo^SD^ is not different from distribution of wild type Robo neither on the longitudinal tracts, nor on the commissures.
(10.49 MB TIF)
