# Supplementary figures and images for: Redundant Mechanisms for Regulation of Midline Crossing in Drosophila
Source: PLoS One. 2008 Nov 24;3(11):e3798. doi: 10.1371/journal.pone.0003798 (PMC2583054; doi:10.1371/journal.pone.0003798)

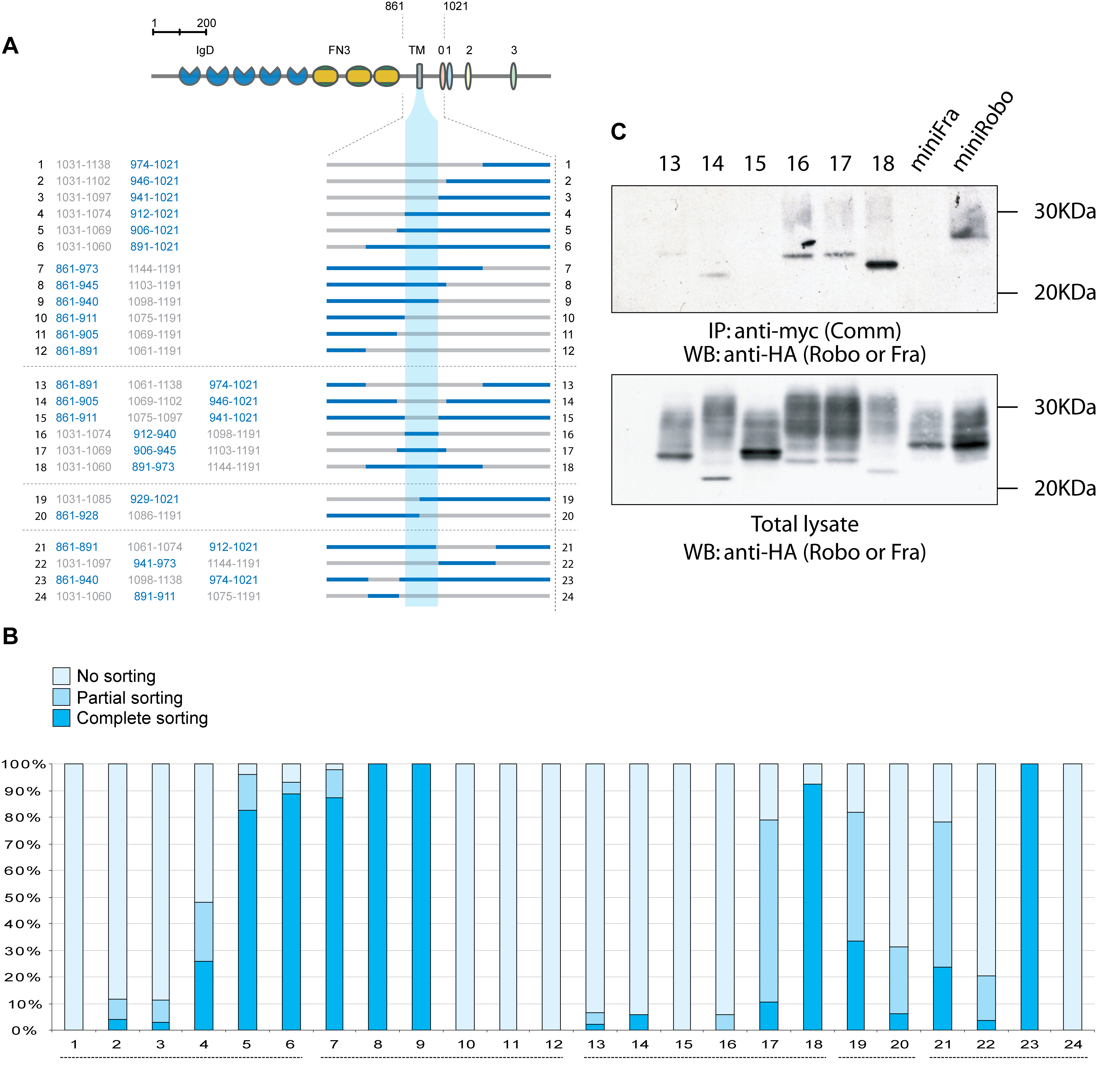

Supplement: Figure S1 — Generation of RoboSD–in vitro analysis of miniRobo chimerical constructs. (A) Schematic representation of the 24 chimerical constructs between the mini-Robo (light blue) and mini-Frazzled (light gray) proteins. The total length of the constructs varies between 255 aa and 268 aa. All constructs have a N-terminal HA tag and a C-terminal V5 tag. Numbers on the left side of each contruct indicate the aa position at which the chimeric ligation occurred. (B) Quantification of sorting activity as observed in the COS-7 cell assay for the 24 constructs listed in (A). (C) Co-immunoprecipitation of Comm with the chimerical constructs 13–18, mini-Robo and mini-Fra. Molecular weight markers are indicated on the right, in kDa. (3.71 MB TIF) [file pone.0003798.s001.tif]

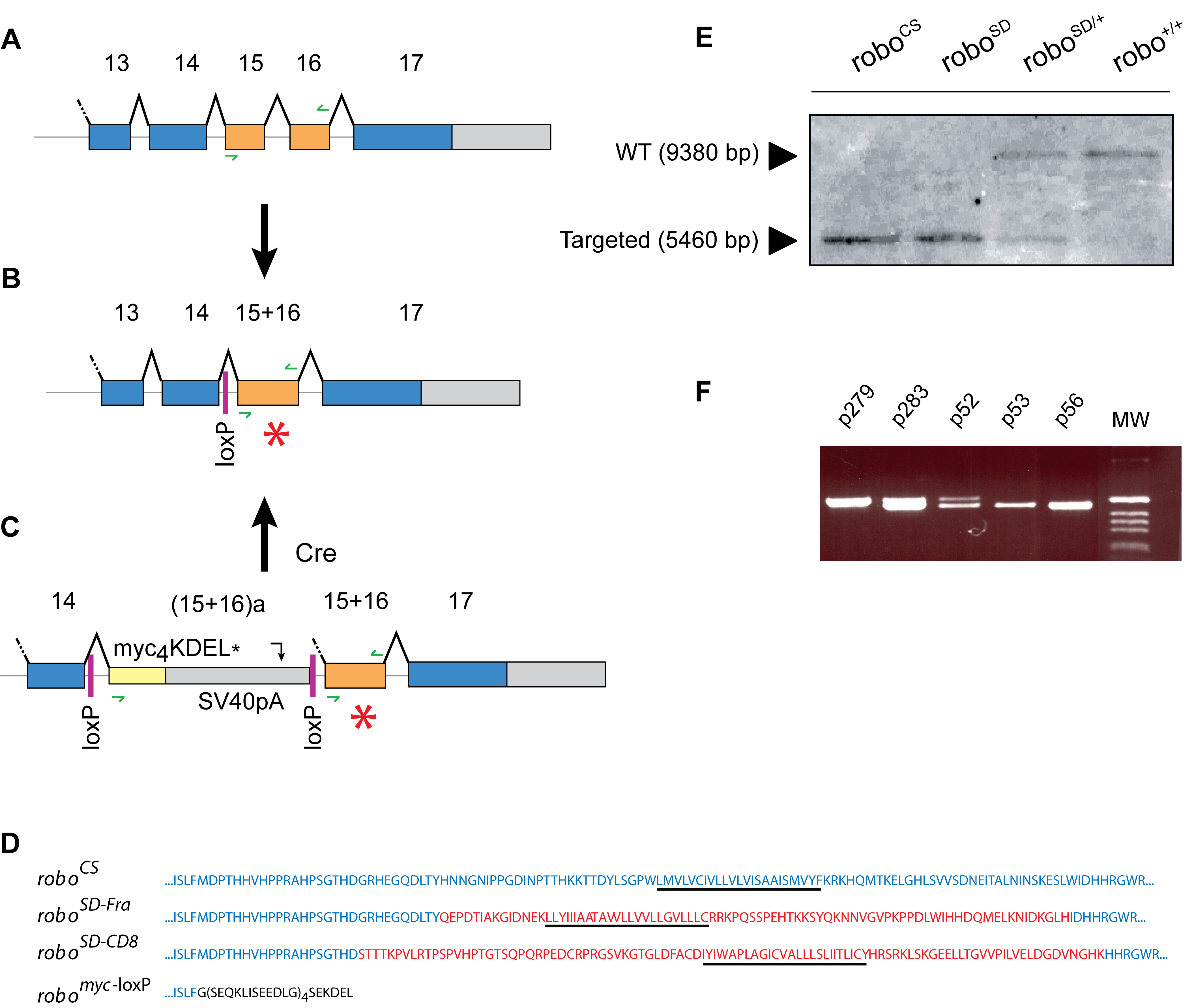

Supplement: Figure S2 — Targeted constructs and molecular validation of recombinant flies. (A–C) Schematics of robo gene structure in wild type and in its modified forms. Blue boxes indicate exons not modified by the gene targeting. Orange boxes indicate modified exons 15 and 16 encoding for the trans- and juxta-membrane domains. Gray boxes indicated untranslated regions. Exons 15 and 16 encode for the peri-membrane region (from 858ISLF to NCRK1032). In the homologous recombinants exons 15–16 have been replaced by one single exon (red asterisk). The newly created gene lacks therefore the intron 15–16 and this allows to easily screen a conspicuous number of lines for proper insertion with PCR amplification using primers sitting on the edges of the two exons (green arrows, see also F). In the conditional modification of Robo (robomyc-loxP, C) a 1 kb sequence precedes the modified exon and prevents the protein from being expressed (see text for details on the sequence). After excision of the loxP cassette, the genomic sequence does not differ from the one of the constitutive alleles in B. (D) Sequences of the modified proteins in the region translated by the new artifical exon 15+16. Blue indicates the original Robo sequence; red indicates the original Fra or CD8 sequences; the predicted transmembrane domains are underlined. In RoboSD-Fra the sequence of Robo comprised between aa 891HNNG and ESLW973 is replaced by the sequence of Fra from aa 1061QEPD to KGLH1143. In RoboCD8-Fra the sequence of Robo comprised between aa 881GRHE and LWID975 is replaced by the sequence of mouse CD8 comprised between aa 151STTT and RSRK224 plus a 26 aa linker sequence derived from a mCD8-GFP fusion protein. (Robo = gi 2804782, Frazzled = gi 24653090, mCD8 = gi 1049227). Robomyc-loxP is the protein that is produced in the conditional alleles, ending at aa ISLF861 and terminating with four copies of a myc tag and a ER retention sequence (KDEL [23]) to avoid secretion of a potential dominant negative form. (E) South [file pone.0003798.s002.tif]

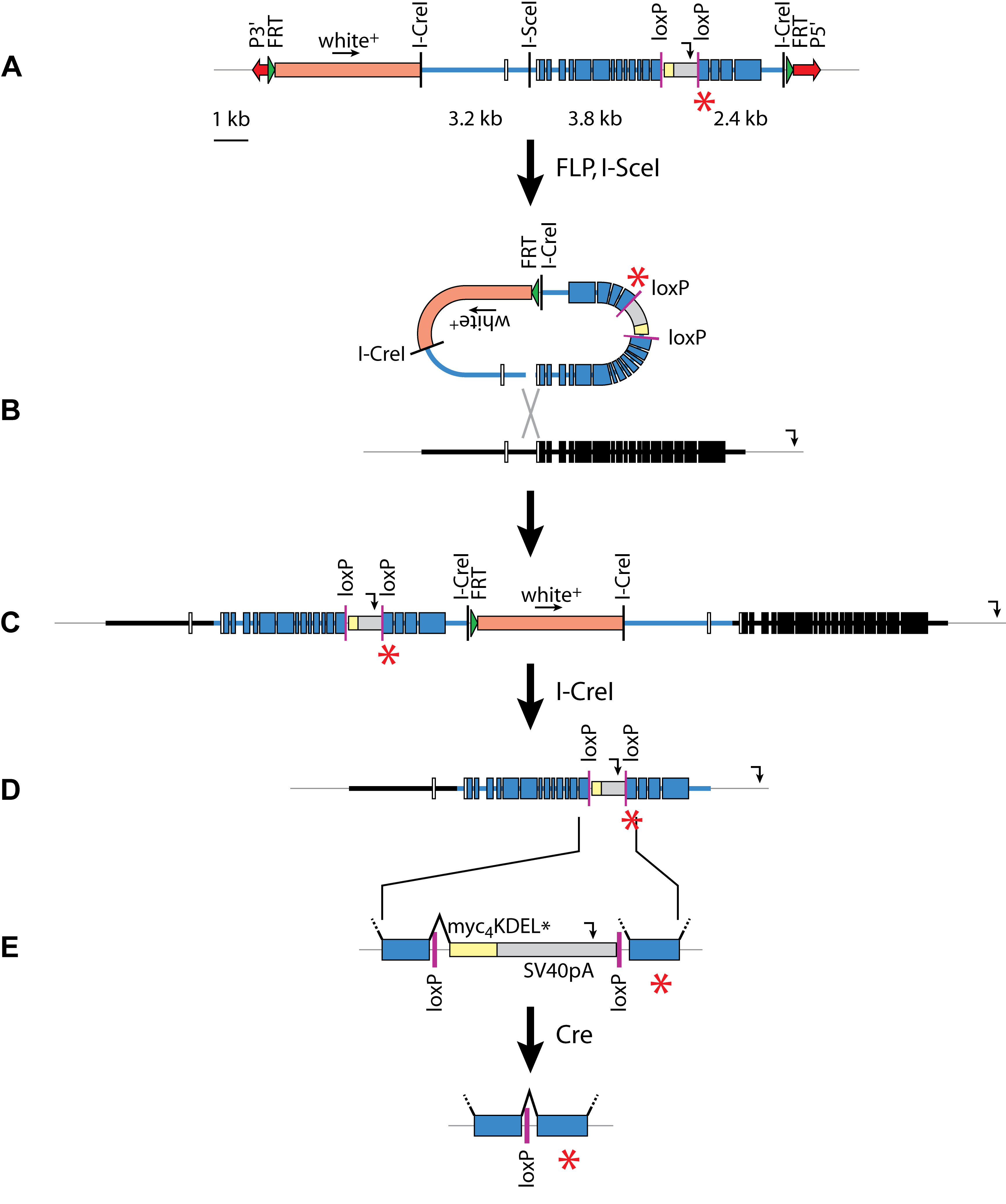

Supplement: Figure S3 — Schematics showing the structure of a construct used to generate a RoboSD allele by homologous recombination. The flox-able version of RoboSD-Fra as an example of a targeting construct used in this work. (A) Successfully transformed flies have the entire targeting construct randomly inserted in the genome. (B) These flies are crossed to ey-FLP, I-SceI expressing flies to excise the targeting construct and start the process of homologous recombination. (C) If the targeting by homologous recombination is successful, the targeted region is modified with a partial duplication of the gene and the inclusion of the modified form. (D) Crossing this targeted flies to I-CreI expressing flies will excise a linear fragment of DNA and resolve the duplication removing also the white mini-gene used as marker for transgenesis. (E) In case of the conditional alleles, the Cre enzyme is used to remove a loxP cassette. (1.03 MB TIF) [file pone.0003798.s003.tif]

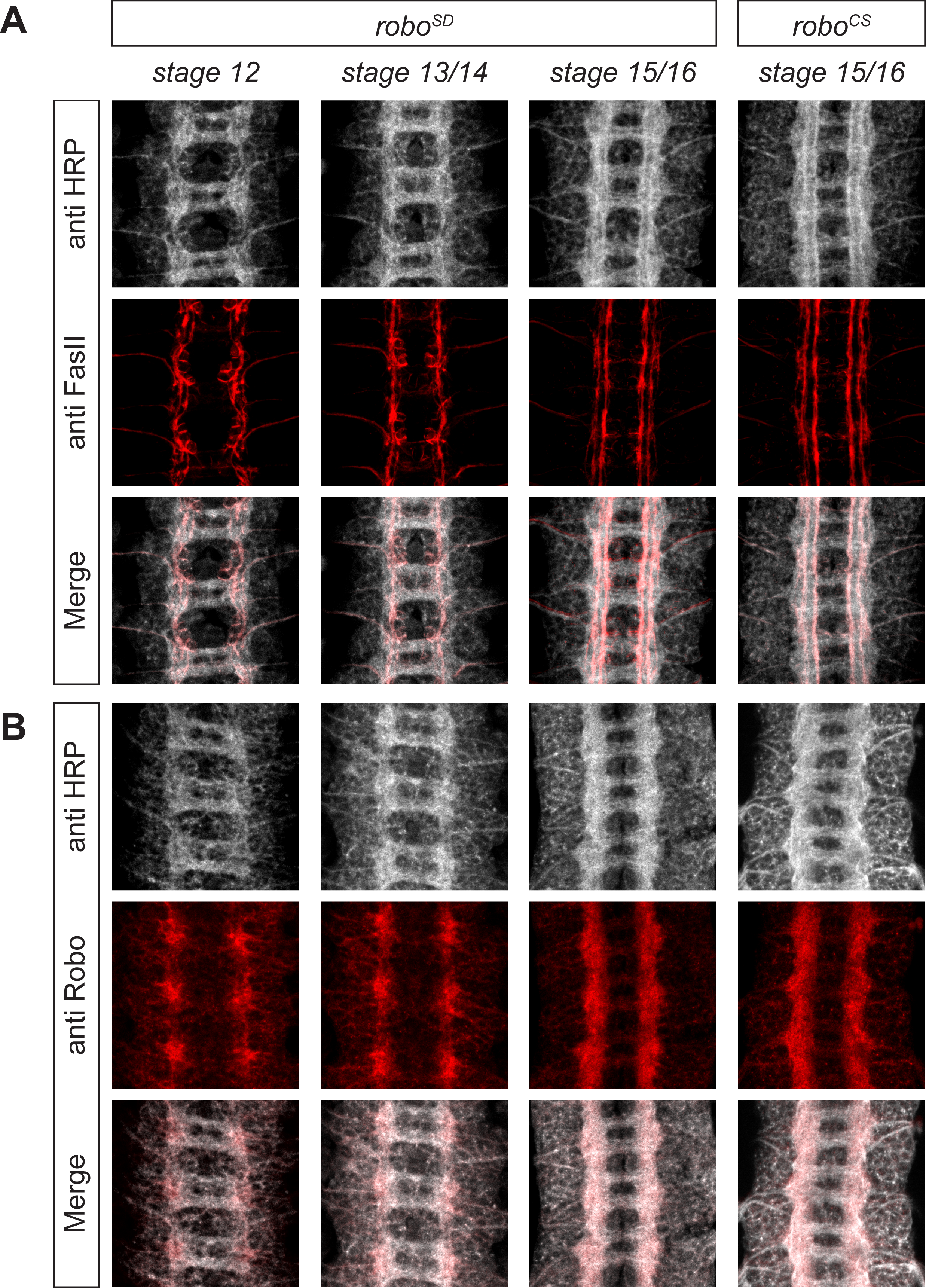

Supplement: Figure S4 — Phenotypic analysis of roboSD embryonic CNS. (A) Representative pictures of a wild type CNS at all stages of development in roboSD embryos (left three columns) and roboCS (wild type control) stage 15 embryos (rightmost column). Embryos were stained using anti-HRP antibody (upper row, gray), directed against a pan-neuronal marker and labeling the entire nervous system and anti FasII antibody (ID4, middle row, red) labeling three longitudinal fascicles running along the longitudinal line. Bottom row provides a merge of both channels. (B) Distribution of the RoboSD protein during development (left three columns) and RoboCS (rightmost column) at stage 15. Distribution of RoboSD is not different from distribution of wild type Robo neither on the longitudinal tracts, nor on the commissures. (10.49 MB TIF) [file pone.0003798.s004.tif]
